# PDE7B Is a Novel, Prognostically Significant Mediator of Glioblastoma Growth Whose Expression Is Regulated by Endothelial Cells

**DOI:** 10.1371/journal.pone.0107397

**Published:** 2014-09-09

**Authors:** Michael D. Brooks, Erin Jackson, Nicole M. Warrington, Jingqin Luo, Jason T. Forys, Sara Taylor, Diane D. Mao, Jeffrey R. Leonard, Albert H. Kim, David Piwnica-Worms, Robi D. Mitra, Joshua B. Rubin

**Affiliations:** 1 Department of Pediatrics, Washington University School of Medicine, St. Louis, Missouri, United States of America; 2 BRIGHT Institute, Washington University School of Medicine, St. Louis, Missouri, United States of America; 3 Molecular Imaging Center, Mallinckrodt Institute of Radiology, Washington University School of Medicine, St. Louis, Missouri, United States of America; 4 Division of Biostatistics, Washington University School of Medicine, St. Louis, Missouri, United States of America; 5 Department of Neurosurgery, Washington University School of Medicine, St. Louis, Missouri, United States of America; 6 Department of Cell Biology & Physiology, Washington University School of Medicine, St. Louis, Missouri, United States of America; 7 Department of Cancer Systems Imaging, University of Texas MD Anderson Cancer Center, Houston, Texas, United States of America; 8 Department of Genetics and Center for Genome Sciences and Systems Biology, Washington University School of Medicine, St. Louis, Missouri, United States of America; 9 Department of Anatomy and Neurobiology, Washington University School of Medicine, St. Louis, Missouri, United States of America; University of Florida, United States of America

## Abstract

Cell-cell interactions between tumor cells and constituents of their microenvironment are critical determinants of tumor tissue biology and therapeutic responses. Interactions between glioblastoma (GBM) cells and endothelial cells (ECs) establish a purported cancer stem cell niche. We hypothesized that genes regulated by these interactions would be important, particularly as therapeutic targets. Using a computational approach, we deconvoluted expression data from a mixed physical co-culture of GBM cells and ECs and identified a previously undescribed upregulation of the cAMP specific phosphodiesterase PDE7B in GBM cells in response to direct contact with ECs. We further found that elevated PDE7B expression occurs in most GBM cases and has a negative effect on survival. PDE7B overexpression resulted in the expansion of a stem-like cell subpopulation *in vitro* and increased tumor growth and aggressiveness in an *in vivo* intracranial GBM model. Collectively these studies illustrate a novel approach for studying cell-cell interactions and identifying new therapeutic targets like PDE7B in GBM.

## Introduction

Studies of tumor biology frequently focus on the intrinsic properties of cancer cells, such as their growth rate, signaling cascades, or DNA repair capacity, without fully accounting for how the microenvironment influences these functions. Tumor progression, however, is a collaboration between the genomic lesions in tumor cells and alterations in the tumor microenvironment [1]. The tumor microenvironment is highly heterogeneous [2] with varying cellular constituents within multiple tumor microdomains such as the leading edge of invasion and perinecrotic or perivascular spaces. Within each of these microdomains, genetically identical tumor cells may exhibit different patterns of gene and protein expression, resulting in regions of distinct cellular phenotypes being simultaneously present within the same tumor. This intratumoral heterogeneity, both phenotypic and genetic, creates a significant experimental challenge in studying cancer biology [3].

Several cancers have been reported to display substantial intratumoral heterogeneity, including glioblastoma (GBM), the most common malignant primary brain tumor in adults. While the study of perinecrotic and invasive edge biology in GBM has generated insights into the metabolic adaptations of cancer cells to hypoxia [4], Notch signaling [5], and the importance of matrix metalloproteinases (MMPs) [6], it is the focus on the biology of the perivascular niche (PVN) that has yielded the greatest body of information. The PVN is home to a subpopulation of tumor cells with stem cell-like properties. The GBM PVN contains GBM cancer stem cells (CSCs), ECs, pericytes [7], astrocytes [8], and microglia [9]. While multiple pathways have been identified as essential for the specialized functions of the PVN [10], [11], how this specialized domain is established remains largely unknown. It is clear that ECs within the GBM PVN are distinct from ECs in the normal brain and that tumor cells within the perivascular space are distinct from bulk tumor cells [10], [12]. Identifying the mediators and targets of these reciprocal interactions will be essential for understanding and effectively targeting PVN function.

Previously, we reported an *in vitro* model of the GBM PVN comprised of primary cultures of human brain microvascular endothelial cells (HBMECs) on Matrigel co-cultured with either an established GBM cell line (U87-MG) or primary GBM cells [13]. Functional studies using this system revealed that expression of the chemokine CXCL12 by HBMECs promoted localization of GBM cells to the peri-endothelial cell space and triggered their expansion. These studies demonstrated the utility of an *in vitro* co-culture system for modeling GBM-PVN interactions. Here we sought to use this system to identify the pathways that are modulated by interactions between endothelial and GBM cells.

## Results

### Global expression profiling identifies genes regulated by GBM cell-EC interactions

We previously demonstrated that the physical co-culture of primary HBMECs and either primary human GBM cells or the U87 GBM cell line resulted in EC-dependent growth of the GBM cells [13]. These findings suggested that the co-culture model faithfully captured an element of GBM tissue biology and could be used for studying the pathways that mediate the tumor promoting effects of ECs on GBM cells. To further validate the biological relevance of the co-culture model, we investigated whether it also recapitulated the effects of tumor cells on ECs. GBM is highly angiogenic and this effect is dependent upon tumor cell secretion of angiogenic factors. We used the publically accessible WimTube image analysis tool (Wimasis.com) and quantified the *in vitro* angiogenic effect of U87 cells on HBMECs. HBMECs were engineered to express mCherry fluorescent protein and cultured in Matrigel as previously described [14]. U87 cells were plated onto the HBMEC tubules 24 hours later, and angiogenesis was assayed in images of mCherry fluorescence as total area covered by ECs, total number of EC tubule loops, and total EC tubule length, every 24 hours for three days in U87-EC co-cultures and in control EC monocultures. We found that U87 cells stimulated significant increases in all three measures of *in vitro* angiogenesis (**Figure S1**). Together with our published findings, these new data indicated that reciprocal interactions relevant to tumor tissue biology occur between GBM cells and ECs in the co-culture model.

**Figure 1:**
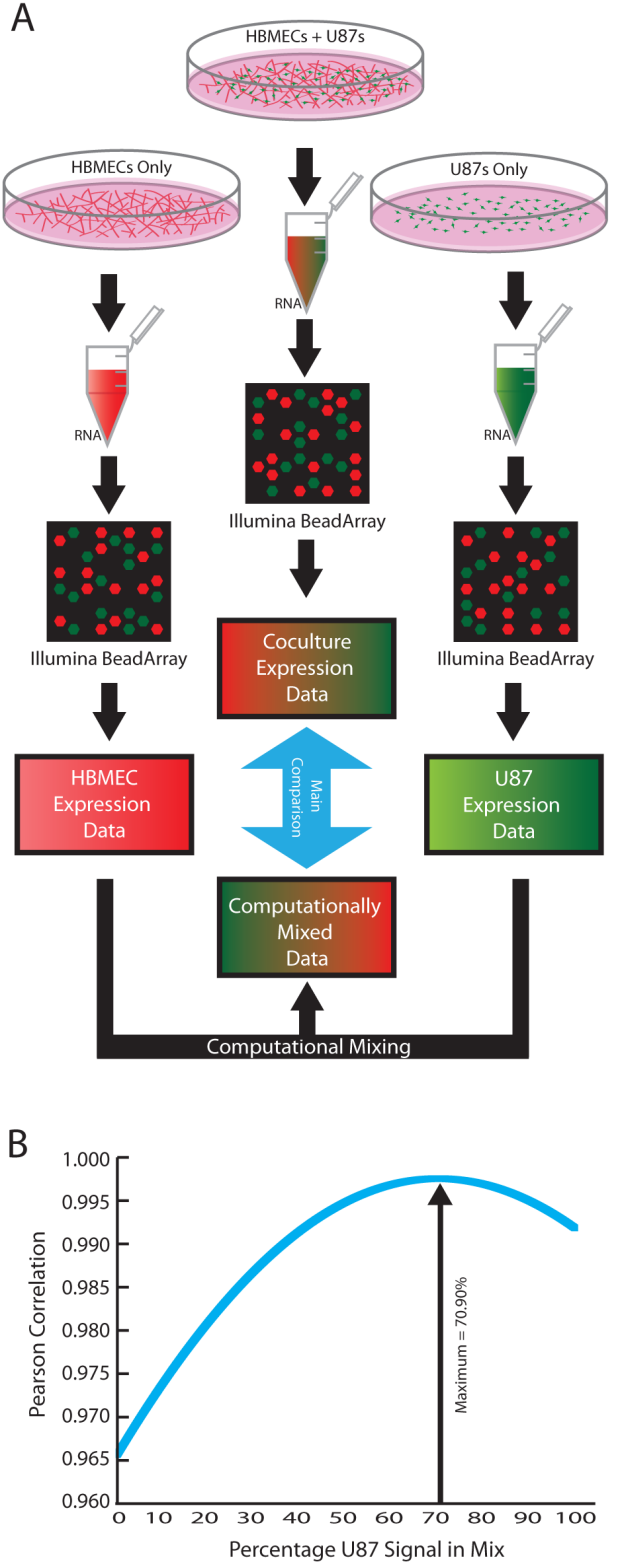
Sample preparation and computational deconvolution of the mixed culture RNA signal. (**A**) Flow chart of sample preparation from culture to gathering RNA and computational analysis. (**B**) The percentage of each monoculture to use for the control dataset was determined by creating one thousand test datasets, each with a different amount of U87 or HBMEC signal, and then calculating the Pearson correlation coefficient between each of these datasets and the measured co-culture dataset.

To identify targets and mediators of the reciprocal interactions between tumor cells and their microenvironment, we performed gene expression profiling with Illumina BeadArray microarrays using RNA isolated from the mixed cell population after 48 hours of culture and RNA isolated from parallel monocultures of HBMECs and U87 cells (**Figure 1A**). Because the RNA isolated from the co-cultures was derived from a mixed population of cells, measured changes in gene expression could occur as a consequence of changes in the relative numbers of HBMECs and U87 cells over the 48-hour co-culture time period, and/or as a result of changes in gene expression induced through functional interactions between the two cell types. As we were interested only in the latter, it became essential to develop computational methods to distinguish between these sources of change.

To accomplish this, we developed an approach that uses gene expression data to precisely determine the ratio of HBMEC and U87 cells within a mixed culture. Previously published global expression profiling of cell-cell interactions has found that less than 10% of genes exhibit statistically significant differential expression upon co-culture [15], [16]. Thus, we anticipated similar results and assumed that the expression levels of most genes in either cell type would remain unchanged upon co-culture, and that only a small percentage of genes would be affected by cell-cell interactions. Using expression profiling data obtained separately from HBMEC and U87 monocultures, we created one thousand computationally mixed datasets for the two cell types, from a ratio of 0.1% HBMEC/99.9% U87 to a ratio of 99.9% HBMEC/0.1% U87 in 0.1% increments. We then used these computed profiles to determine the precise ratio of GBM and HBMECs in the co-culture at the time of mRNA isolation. To do so, we calculated the Pearson correlation coefficient between the experimentally measured co-culture expression data and the full series of computationally generated expression profiles. We concluded that the synthetic profile with the highest correlation to the measured co-culture profile provided the closest approximation to the actual ratio of U87 cells and HBMECs in the co-culture (**Figure 1B**). This calculation was performed for three independent sets of co-culture data. In each case, normalization of the co-culture profile to the synthetic profile with the highest correlation identified those transcripts whose level of expression differed from the norm. These transcripts represented the candidate genes whose expression was either increased or decreased through functional interactions between HBMECs and U87 cells (for further information, see Methods S1).

In this manner, 45 genes with at least a two-fold change in expression were identified as significantly (after Benjamini-Hochberg multiple hypothesis correction) upregulated or downregulated as the result of functional interactions between HBMECs and U87 cells (**Table 1**). Consistent with known effects of GBM cells on ECs and our results showing an increase in *in vitro* angiogenesis upon co-culture (**Figure S1**), this list of genes contained several regulators of angiogenesis such as Thrombospondin-1 [17] and CXCL1 [18]. Also notable is the upregulation of WNT signaling genes CTHRC1 [19] and Frizzled-9 [20]. WNT signaling is known to be involved in maintaining stemness and cell proliferation [21].

**Table 1:** Genes differentially expressed by co-culture of U87 and HBMECs.

Symbol	Corrected p-value	Fold Change	Symbol	Corrected p-value	Fold Change
PRRX2	3.25E-04	4.43*	CD36	3.25E-04	−2.37
CTHRC1	0.013	2.06	MBP	7.72E-03	−2.95
FILIP1L	0.015	2.86	LOC100131643	8.21E-03	−2.85
C8orf4	0.015	2.04	KIAA1199	0.0082	−2.64
PDE7B	0.020	2.20	DLX5	0.0082	−2.42
SOBP	0.020	2.19	MBTD1	0.0084	−2.15
MTRR	0.022	8.83	KIAA0692	0.015	−9.23
ICAM1	0.022	4.56	GREM1	0.020	−2.00
CXCL1	0.022	4.51	MIR204	0.024	−18.27
A4GALT	0.022	3.84	ZFAT	0.024	−2.52
MSI2	0.022	2.49	ZXDA	0.024	−2.23
MXRA5	0.022	2.33	MIR2117	0.026	−3.34
FAM179A	0.022	2.24	ZMYM3	0.028	−20.38
FZD9	0.026	12.33	KLK11	0.029	−3.40
MED15	0.026	2.24	DIRC1	0.032	−2.10
LOC650566	0.028	2.73	HS3ST2	0.032	−2.27
FNDC5	0.029	27.45	THBS1	0.033	−2.58
CDKN2C	0.032	22.06	C9orf96	0.033	−2.29
CCL20	0.034	2.68	EGR3	0.034	−3.00
PNLIPRP3	0.044	2.25	LOC285501	0.034	−2.47
SLCO2A1	0.046	10.51	RCAN2	0.040	−3.04
LOC729666	0.047	3.22	CD80	0.048	−2.30
C9orf150	0.048	2.20			

*All genes with a Benjamini-Hochberg corrected p-value<0.05 and greater than a two-fold change are shown. Fold change was calculated as: (Co-culture dataset/Computational dataset) for genes with increased expression upon co-culture, or as: −(1/(Co-culture dataset/Computational dataset)) for genes downregulated.

### Validation of expression profiling results by conditioned media swaps

**Figure 2:**
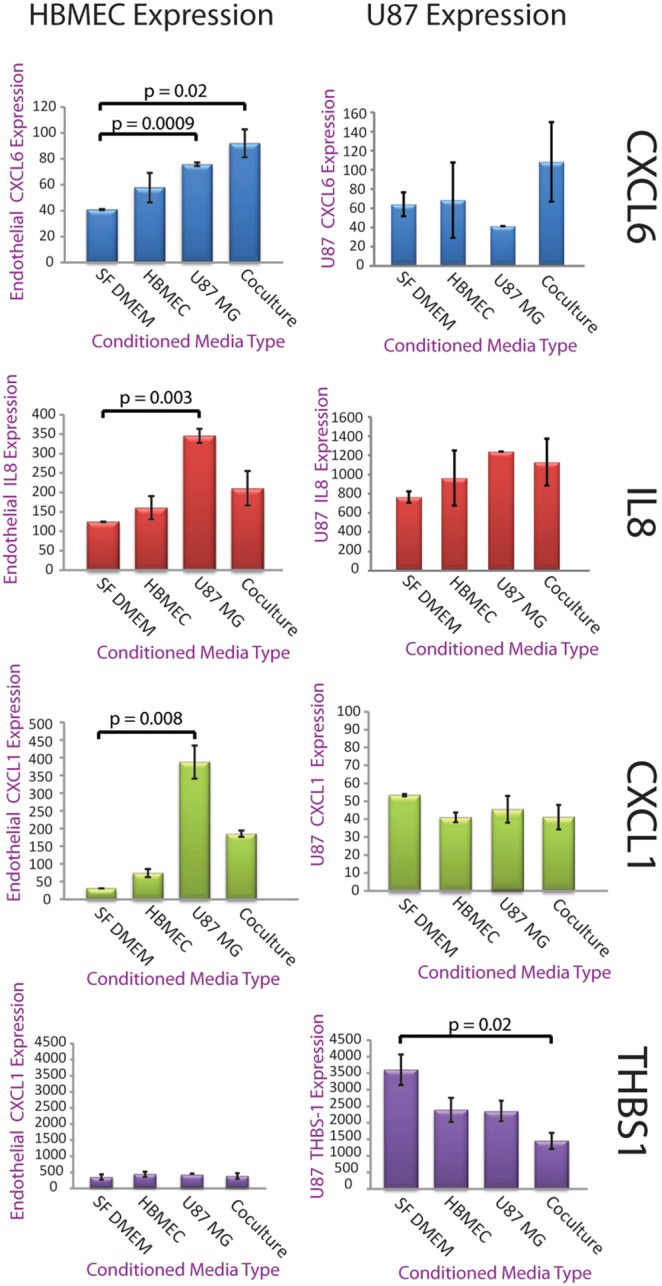
Specification of the cell of origin for expression changes by qRT-PCR validation of differentially expressed genes using conditioned media swap. Conditioned medias were Serum Free DMEM (SF DMEM), SF DMEM conditioned by HBMECs (HBMEC), SF DMEM conditioned by U87 cells (U87 MG), or SF DMEM conditioned by a co-culture of HBMECs and U87s. HBMECs (left column) or U87s (right column) were grown in one of the respective conditioned media types for 48 hours, RNA was isolated, and qRT-PCR was performed for CXCL6, CXCL1 and THBS1. Expression of CXCL6 and CXCL1 specifically changed in the HBMECs in response to U87 or co-culture CM, while changes in THBS1 expression were limited to the U87 cells and occurred most significantly in response to co-culture CM. Expression is in arbitrary quantification units calculated by performing standard delta-delta C(t) while setting GAPDH expression to an arbitrary level of 10,000. N = 3 for all sets, significance by Student’s t-test.

The analysis thus far identified genes whose expression changed as a result of cell-cell interactions, but did not identify the cells in which these changes were occurring or whether the changes were in response to the actions of secreted factors or direct cell-cell contact. To establish requirements for contact and to identify the target cell for expression changes, we performed monoculture experiments in which ECs and U87 cells were treated with either basal media, media conditioned by U87 or EC monocultures, or by U87-EC co-cultures. We took advantage of the robust angiogenic effect of co-culture to validate the approach. Addition of U87 cells to the HBMEC tubules promoted *in vitro* angiogenesis (**Figure S1**) and the expression of angiogenesis regulators: CXCL1, and THBS1 were both altered by co-culture (**Table 1**). Thus we evaluated expression changes for CXCL1 and THBS1 and as well as for CXCL6, another angiogenesis related chemokine. In the main computational analysis, changes in expression for CXCL6 did not pass multiple hypothesis correction, but based on its relevance to angiogenesis and nearly significant p-value of 0.053 it was included in the analysis. For all three genes, conditioned media (CM) treatments recapitulated the gene expression changes predicted from the microarray results indicating that their regulation was through the actions of secreted factors (**Figure 2**). CXCL6 expression was observed in both HBMECs and U87 cells under basal conditions. While CXCL6 expression in U87 cells was unaffected by any of the CM preparations, it was significantly increased in HBMECs in response to either U87 or co-culture CM. These data indicated that changes in expression of these transcripts occurred in the EC compartment in response to factors secreted by U87 cells. CXCL1 was expressed at low levels in both U87 cells and HBMECs under basal conditions. Similar to CXCL6, CXCL1 expression in U87 cells was unaffected by any of the CM but significantly increased in HBMECs in response to U87 and co-culture CM. In contrast to these other factors, THBS1 expression was strikingly higher in U87 cells under basal conditions and its expression was strongly downregulated by all CMs. These results validated the computational and experimental approach and indicated that conditioned media experiments could distinguish the regulator and target cell type for gene expression changes that occurred through the actions of secreted factors during GBM and EC interactions.

### GBM PDE7B expression is regulated by physical contact with HBMECs

To prioritize transcripts for further validation, we considered the magnitude of expression changes induced through cell-cell interactions as well as whether the transcripts had potentially novel and important roles in GBM biology. Among the genes upregulated by HBMEC-U87 cell interactions was the cAMP specific phosphodiesterase PDE7B [22]. This transcript was of particular interest as we previously demonstrated that another cAMP-specific phosphodiesterase, PDE4A1, had potent pro-tumorigenic effects in both low and high-grade glioma models [23], [24]. Thus we hypothesized that if PDE7B were upregulated in GBM cells, it might also stimulate GBM growth, and its catalytic activity might constitute a novel therapeutic target, similar to other cAMP specific phosphodiesterases [24], [25], [26].

**Figure 3:**
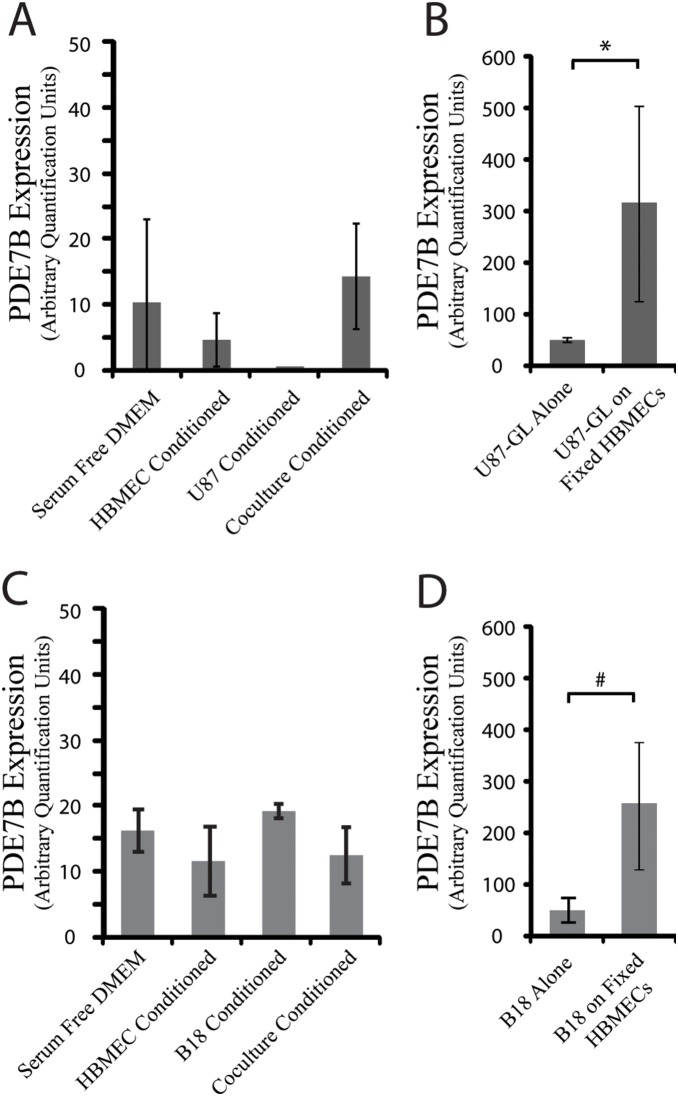
Changes in PDE7B Expression require physical contact between endothelial cells and U87 cells. (**A**) PDE7B expression was measured by qRT-PCR in RNA isolated from U87 cells grown alone or on methanol fixed HBMECs. *p-value = 0.04 by Student’s t-test. (**B**) PDE7B expression similarly measured in RNA isolated from a primary GBM line, B18, grown alone or on fixed HBMECs. #p-value = 0.02 by Student’s t-test.

PDE7B expression was unaffected in media swap experiments (**data not shown**), therefore we suspected it might be regulated by direct cell-cell contact. To test this hypothesis, HBMECs at ∼80% confluence were methanol fixed and washed. These fixed cells and their associated matrix, maintain relevant epitopes for cell-cell and cell-matrix interactions but are devoid of secreted factors and RNA [27]. U87 cells were then cultured on the fixed HBMECs for 48 hours prior to RNA extraction. Under these conditions, PDE7B was upregulated in the U87 cells, indicating that in contrast to the regulators of angiogenesis previously evaluated, changes in U87 PDE7B expression only occur through physical cell-cell contact with HBMEC cells (**Figure 3A**). To verify that HBMEC-induced changes in PDE7B were not confined to U87 cells and to increase the relevance of these findings to GBM, we performed an identical evaluation with a primary low passage GBM cell line B18. Consistent with the results obtained with U87 cells, PDE7B was upregulated in B18 cells, but only through direct physical contact (**Figure 3B**).

### PDE7B is frequently upregulated in GBM and has prognostic significance

**Figure 4:**
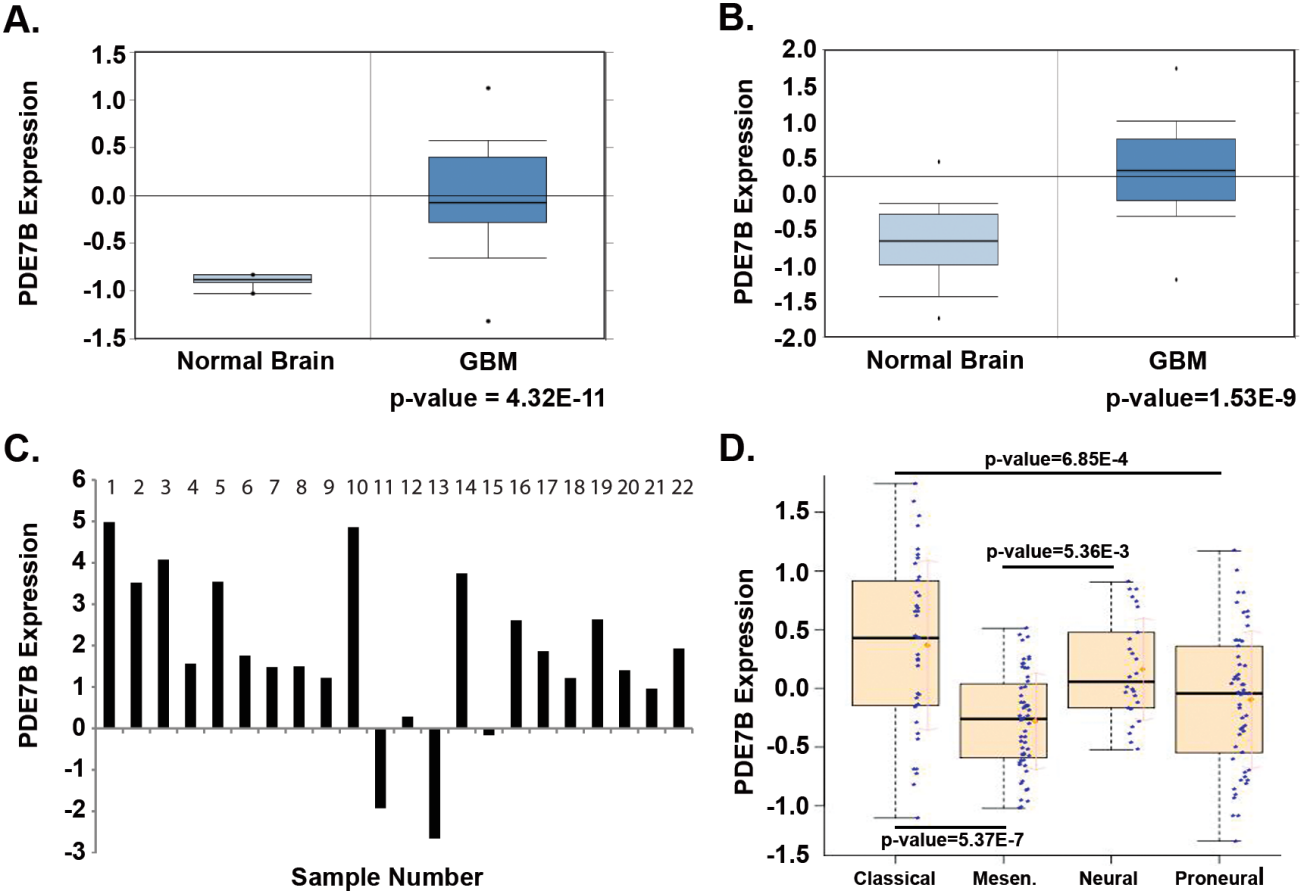
PDE7B is expressed in human GBM. (**A**) Oncomine Murat et al [28] dataset showing overexpression of PDE7B in GBM samples compared to normal brain. (**B**) Oncomine Sun et al [29] dataset also showing overexpression of PDE7B in GBM samples compared to normal brain. (**C**) cDNA was prepared from twenty-two primary human GBM RNA samples. PDE7B expression was quantified by qRT-PCR and compared to expression in normal human astrocytes. (**D**) Relationship between PDE7B expression and molecular subtypes of GBM, *Classical* (C), *Mesenchymal* (M), *Proneural* (P) and *Neural* (N). Shown are box and whisker plots of data accessed through the TCGA as described in Materials and Methods. P-values for differences in subtype specific expression are shown.

To establish overall PDE7B expression in GBM, we first examined two independent publically accessible gene expression datasets. Using Oncomine (www.oncomine.org) we found datasets by both Murat et al [28] and Sun et al [29] showing that PED7B expression was significantly greater in GBM compared to normal brain controls ((p = 4.23E-11, p = 1.53E-9, respectively, **Figure 4A, B**). We next verified PDE7B over expression by qRT-PCR in 18 of the 22 primary GBM specimens (**Figure 4B**). Finally, we looked for subtype specific patterns of PDE7B expression using data from The Cancer Genome Atlas and molecular subtype-characteristic centroid expression profiles as previously described by us [30]. PDE7B expression was significantly greater in the *Classical,* compared to *Mesenchymal* (P = 5.37E-07) or *Proneural* (P = 6.85E-04) subtypes of GBM (**Figure 4D**). There was also a significant difference between PDE7B expression in *Mesenchymal* and *Neural* subtypes of GBM (P = 5.36E-03). Together these data indicate that PDE7B mRNA expression is increased in GBM and that PDE7B expression may distinguish between subtypes of disease.

**Figure 5:**
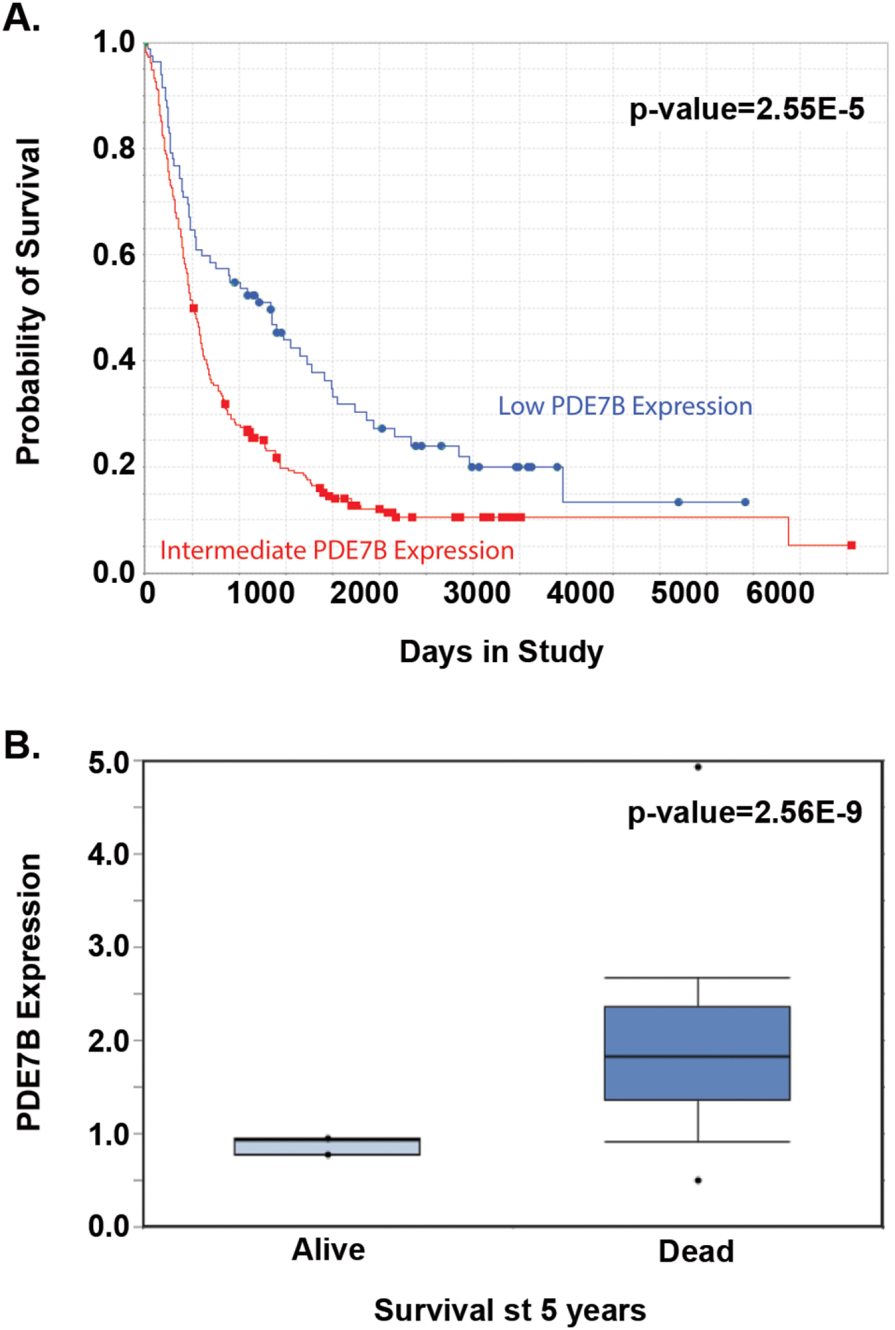
PDE7B expression is correlated with Survival. (**A**) Data from NCI’s Rembrandt database indicates that expression of PDE7B at levels two-fold lower than median is strongly correlated with improved survival compared to median levels of expression in all glioma samples. p-value = 2.55E-5 (**B**) Oncomine’s Murat et al [28] dataset showing that in GBM patients those who were alive at 5 years had very much lower levels of PDE7B than those who had succumb to disease at that point (p-value = 2.56E-9).

To determine whether levels of PDE7B expression are prognostic in GBM we first examined the National Cancer Institute’s (NCI) Rembrandt Database for all grades of astrocytoma. Stratification of all astrocytomas based on PDE7B expression two-fold above or below the median level of expression revealed a striking correlation with survival. While too few cases with PDE7B expression greater than two-fold above the median were available for survival analysis, PDE7B expression 2-fold lower than the median was correlated with significantly increased survival compared to median levels of expression (p-value = 2.55E-5) (**Figure 5A**). The effect of PDE7B on survival was distinct from that of two other cAMP specific phosphodiesterases evaluable in the Rembrandt Database. Expression of PDE4A and PDE8B at two-fold lower than the median was correlated with worse survival, while expression of PDE8B at two-fold higher than the median was associated with better survival, compared to cases with median levels of expression (**Figure S2**). This analysis suggests there may be some specificity to the actions of PDE7B in GBM. We confirmed the effect of PDE7B expression on survival in the Murat et al dataset [31] where patients with GBM alive at 5-years showed significantly lower levels of expression than patients who had died of disease (**Figure 5B**).

### Overexpression of PDE7B stimulates in vitro stemness and in vivo GBM growth and tumorigenicity

To directly test whether the level of PDE7B expression affects GBM biology, we cloned and overexpressed PDE7B in U87-GL cells and a low passage primary GBM cell line, G144. As a control, we engineered a catalytically inactive form of PDE7B. Catalytically inactive forms of other cAMP specific phosphodiesterases have been generated by mutating an essential histidine in the catalytic site [23], [32]. Using comparative genomics we aligned the sequences of these phosphodiesterases with PDE7B, identified the same essential histidine and generated an analogous catalytically inactive histidine to glutamine (H217Q) mutant. Both the wild type and mutant forms of PDE7B were stably introduced using PiggyBac transposase and hygromycin selection. Verification of the overexpression was determined by qRT-PCR (**Figure S3A**), and Western blot analysis (**Figure S3B**). The effect of wild type and mutant PDE7B on cAMP levels was verified by cAMP ELISA. Overexpression of wild type PDE7B, but not catalytically inactive PDE7B, resulted in significantly lower levels of cAMP (**Figure S3C**).

**Figure 6:**
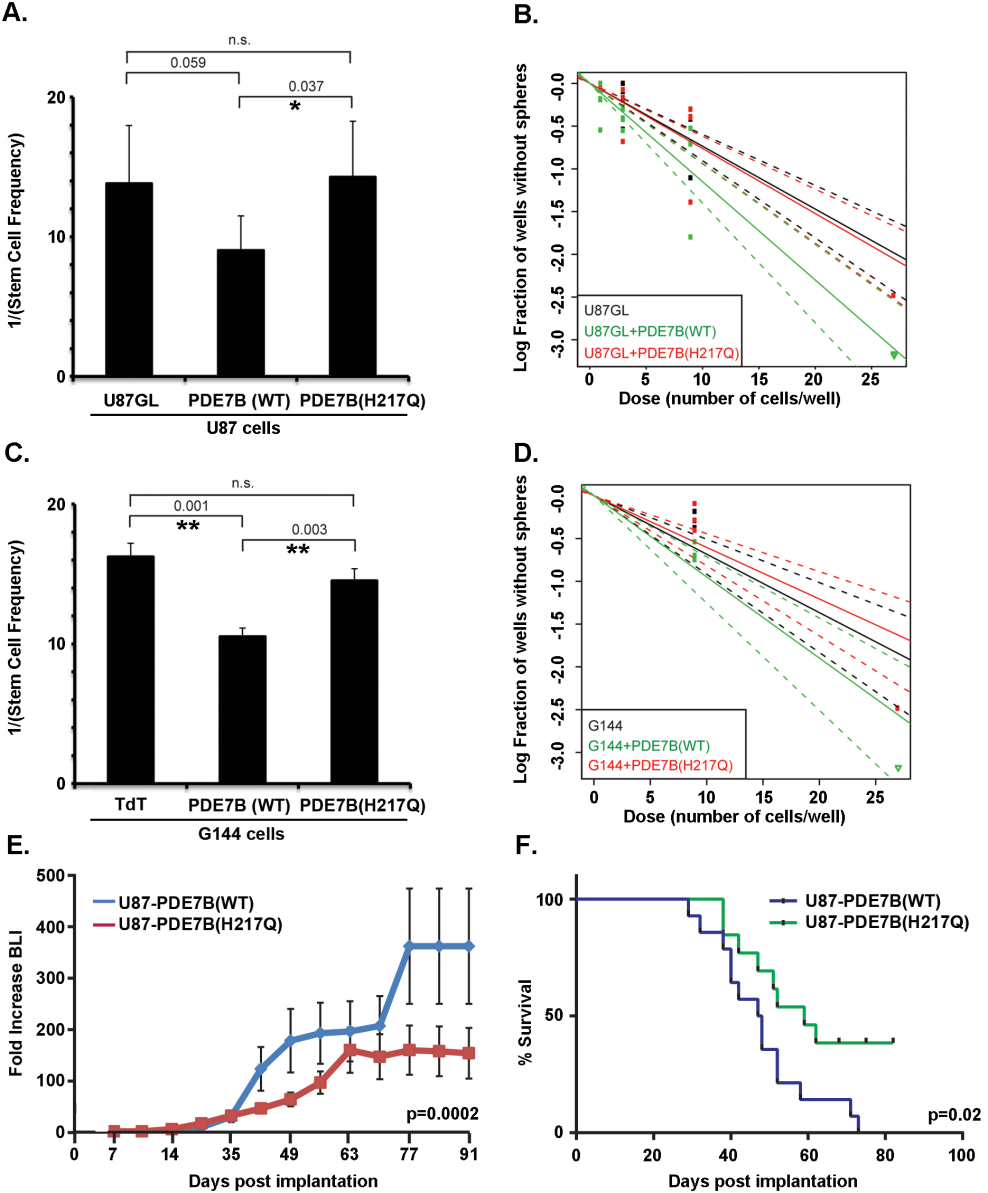
Overexpression of PDE7B increases stem cell fraction and accelerates in vivo tumor growth and decreases survival. (**A**) Frequency of clonogenic stem-like cells as determined by Extreme Limiting Dilution Assays (ELDA) with U87 cells expressing PDE7B (WT), catalytically inactive PDE7B (H217Q) or no vector control (U87GL). PDE7B-WT expression increased the clonogenic cell frequency (*indicates p = 0.037, two tailed t-test) compared to the catalytically inactive form. (**B**) Plot of the log fraction of wells without spheres as a function of plated U87 cell number. The more vertical the line, the higher the percentage of clonogenic, sphere-forming cells. (**C**) Frequency of clonogenic stem-like cells as determined by Extreme Limiting Dilution Assays (ELDA) with G144 cells expressing PDE7B (WT), catalytically inactive PDE7B (H217Q) or empty vector control (TdTomato). PDE7B-WT expression increased the clonogenic cell frequency compared to TdTomato (**indicates p = 0.001, two tailed t-test) and compared to catalytically inactive PDE7B (H217Q) (**indicates p = 0.003, two tailed t-test). (**D**) Plot of the log fraction of wells without spheres as a function of plated G144 cell number. The more vertical the line, the higher the percentage of clonogenic, sphere-forming cells. (**E**) Intracranial growth of luciferase-expressing U87 cells was monitored by weekly bioluminescence imaging (BLI). Data for each individual mouse is normalized to its own week one value and shown are the means and SEM for each group (N = 14 (Wild Type), N = 13 (H217Q)). P-value = 0.0002 as determined by two-way ANOVA. (**F**) Kaplan-Meier plot of survival. Logrank test indicated a significant decrease in survival (p-value = 0.02) in mice xenografted with U87-GL cells overexpressing wild type PDE7B compared to cells overexpressing catalytically inactive PDE7B (N = 14 (Wild Type), N = 13 (H217Q).

As interactions between ECs and GBM cells are known to maintain the GBM stem-like cell state [10], we assayed the effect of PDE7B expression on clonogenic activity in an extreme limiting dilution assay. Briefly, replicate cultures were established with cell numbers that ranged from 1–30 cells/well in sphere formation media and allowed to grow for 3 weeks. Under these conditions, only cultures that contain a clonogenic stem-like cell are capable of forming a sphere. The fraction of wells without spheres as a function of cells plated provides a measure of the frequency of cells with clonogenic stem-like properties (**Figure 6B, 6D**). When analyzed, the U87GL+PDE7B(WT) (**Figure 6A**) and the G144+PDE7B(WT) (**Figure 6C**) cells had significantly higher frequencies of stem-like cells compared to cells expressing the catalytically inactive form of PDE7B. One out of every nine U87GL+PDE7B(WT) cells had sphere-forming ability compared to one out of every 14 U87GL+PDE7B(H217Q) cells. Similarly, one out of every 11 G144+PDE7B(WT) cells had sphere-forming ability compared to one out of every 15 G144+PDE7B(H217Q) cells.

*In vitro* expansion of the stem-like cell population by overexpression of PDE7B, together with the negative prognostic effect of PDE7B expression in human disease, suggested that overexpression of PDE7B might have a pro-tumorigenic effect *in vivo*. To investigate this possibility, we generated intracranial xenografts in NCR nude mice with 50,000 U87-GL+PDE7B(WT) or U87-GL+PDE7B(H217Q) cells engineered to also express firefly luciferase [23]. Tumors were generated in three separate cohorts of mice with 5 mice per group in each cohort. Tumor growth was monitored with weekly bioluminescence imaging (BLI) and survival was tracked. Tumors expressing wild type PDE7B exhibited a significantly greater rate of growth than those expressing mutant PDE7B (**Figure 6E**). The greater rate of growth resulted in significantly decreased survival as determined by log-rank testing of Kaplan-Meier survival curves (**Figure 6F**).

**Figure 7:**
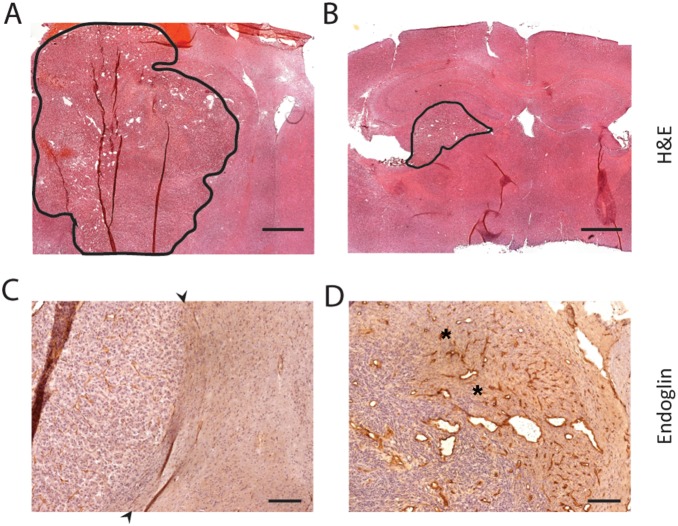
Expression of PDE7B increases tumor vascularity and invasiveness. (**A**) H&E staining of representative U87-GL+PDE7B(WT) tumor, with tumor tissue outlined in black. Scale bar = 1 mm (**B**) H&E staining of representative U87-GL+PDE7B(H217Q) tumor, with tumor tissue outlined in black. Scale bar = 1 mm (**C**) Endoglin staining of a representative U87-GL+PDE7B(H217Q) tumor illustrates usual pattern of U87 growth. Black arrows demarcate tumor-surrounding brain boundary. Tumor cells are not detected in the surrounding brain and there is no angiogenic response in the surrounding brain. Scale bar = 300 µm. (**D**) Immunolabelling for Endoglin of a U87-GL+PDE7B(WT) xenograft revealed a highly invasive tumor leading edge with tumor cells evident in the surrounding brain accompanied by a robust angiogenic response (asterisks). Scale bar = 300 µm.

Consistent with the BLI and survival data, histological analysis revealed that tumor size was consistently greater in the PDE7B WT tumor group (**Figure 7A**) than the PDE7B H217Q tumor group (**Figure 7B**). Strikingly, PDE7B WT tumors exhibited a more aggressive tumor phenotype than is normally seen with U87 xenografts [33]. Intracranial xenografts of U87 cells typically grow in a simple expansile fashion, without invasion of the surrounding tissue, resulting in compression of the surrounding brain. This pattern of growth is evident in the analysis of PDE7B H217Q tumors where a clear boundary is evident between the tumor and the surrounding brain (**Figure 7C**). In contrast, PDE7B WT tumors grew with extensive infiltration. This was clearly evident upon examination of the leading edge of the xenografts where abundant tumor cells can be seen invading the surrounding brain parenchyma accompanied by robust neovascularization (**Figure 7D**).

## Discussion

The complexity of the tumor microenvironment presents obstacles to progress in both understanding molecular oncology and in developing therapeutics to effectively target contextualized tumor biology. Advancement may depend upon new model systems in which the multi-faceted microenvironment can be deconvoluted in order to assess the contributions that specific subsets of cells, matrix, and three-dimensional structures make to tumor tissue biology. Prior efforts to identify the mediators and targets of tumor promoting cell-cell interactions have frequently relied on conditioned media experiments [34], [35], however one drawback to this approach is that changes induced by physical cell-cell interactions or reciprocally induced interactions (feedback loops) cannot be detected. Ideally, model systems like these should support the discovery of all of the genes and pathways that mediate the important reciprocal interactions that occur between tumor cells and their microenvironment [36]. We were interested in understanding the complete set of interactions between GBM and endothelial cells as these are purported to be essential for tumorigenesis [10], tumor progression [37], and recurrence after standard treatment [38]. While the deconvolution of transcriptional data has been previously described [39], [40], [41], here we report a new algorithm tailored for mixed expression signals from two cell types and its application to the novel setting of GBM tumor cells interacting with ECs. Direct global expression profiling of co-cultured GBM and endothelial cells, enabled by our computational deconvolution algorithm, revealed a number of pathways that were induced or suppressed by the interaction of these cells. Among them was upregulation of the cAMP-specific phosphodiesterase, PDE7B, in GBM cells.

This computational approach has its own limitations. First, the detected gene expression changes are a mixture of the signal from both of the constituent cell types. Therefore, for each gene, further studies are required to determine in which of the cell types the change occurred, and whether this change was a function of secreted factors or cell-cell contact. Furthermore, reciprocal gene induction and suppression could theoretically result in no apparent change in signal and a diminution of the model’s sensitivity. We suspect that processes like these contribute to the marginally weaker significance we detected for changes in gene expression as compared to many other global expression studies. However, we were able to identify 45 genes whose expression was significantly altered. After multiple hypothesis correction, validation by qRT-PCR, and *in vivo* experiments we did not uncover any false positives, reassuring us that the analysis provided a high quality list of changes in gene expression as a consequence of functional interactions between GBM and endothelial cells. Currently, the system serves as a proof of principle and evaluates a single set of interactions between two cell types from the tumor microenvironment. Further work will be necessary to systematically add other tumor microenvironmental cells types and determine the additional effects on gene expression.

Of the genes discovered by our assay, PDE7B was chosen for further studies. This focus was supported by our previous work demonstrating that PDE4A1 and cAMP suppression can contribute to tumorigenesis [23] and drive intracranial brain tumor xenograft growth [26] [24]. Low levels of cAMP have been correlated with malignant brain tumors for many years [42], but the mechanisms that control these abnormal cAMP levels and their cancer relevant targets are often undefined or remain poorly understood. The cyclic AMP pathway is integral to the regulation of numerous cellular processes including growth and differentiation [43], [44]. While the bulk of cAMP signaling is transmitted by just two mediators, protein kinase A (PKA) and exchange protein activated by cAMP (EPAC), exquisite specificity in cAMP signaling is achieved through subcellular localization and activation [45]. A critical component of this specification is the establishment of defined intracellular pools of differing cAMP levels as a result of highly localized and regulated cAMP degradation through PDEs.

Humans have 11 families of PDEs, three of which are cAMP specific (PDE4, PDE7, PDE8), and each of these families can have multiple gene homologs throughout the genome, and each of these genes can have multiple splicing isoforms [45], [46]. The canonical PDE structure includes a common catalytic domain, an amino terminal subcellular localization motif and regulatory kinase sites and protein-protein interacting domains. Consequently, PDE expression and activation result in the generation of highly specific gradients of intracellular cAMP [47] and the formation of multiprotein signaling complexes that carry out localized functions [48], [49]. This process has been most extensively illustrated for the PDE4 family of cAMP-specific PDEs [50], [51].

While we have yet to define how PDE7B promotes GBM tumorigenecity, there are some likely mechanisms. Cyclic AMP is a recently established regulator of neural stem cell proliferation, differentiation, and survival [52], and it is known that endothelial cells in the PVN support GBM CSCs. Therefore the induction of PDE7B expression in tumor cells localized to the perivascular space may function to promote CSC function. The detection of a modest, but significant, increase in stemness, as measured by our *in vitro* limiting dilution assay, in both G144 and U87 cells overexpressing wild type PDE7B is consistent with this hypothesis. This would also be in line with our observation that PDE7B WT intracranial tumors showed signs of increased invasion and aggressiveness compared to the PDE7B H217Q tumors. The increase in blood vessel density in wild type tumors also suggests that tumor cell PDE7B might function as part of a positive feedback loop for promoting angiogenesis.

Finally, the effect of PDE7B on outcome in GBM and its ability to drive intracranial tumor growth in the U87 model suggests that inhibition of PDE7B should be evaluated as a novel therapeutic target for GBM. We previously demonstrated that targeted inhibition of an alternate cAMP-specific phosphodiesterase, PDE4, had significant anti-brain tumor activity, when tested in a spontaneous model of low-grade glioma and in intracranial xenograft models of GBM and medulloblastoma [23] [24]. Together with the current findings, these results provide a strong rationale for cAMP elevation in the treatment of brain tumors. While there have not been any studies of specific PDE7B inhibitors in the context of cancer treatment, there has been work done evaluating PDE7B inhibition for the treatment of autoimmune disorders [53], [54]. Therefore future work should focus on taking specific inhibitors of PDE7B from the autoimmune studies and determining their efficacy in blocking glioma growth *in vitro* and *in vivo* with the hope of translating these findings to the clinic as quickly as possible.

## Materials and Methods

### Ethics Statement

*Animal studies:* All animals were used in accordance with an Animal Studies Protocol (#20120174) approved by the Animal Studies Committee of the Washington University School of Medicine per the recommendations of the Guide for the Care and Use of Laboratory Animals (National Institutes of Health).

*Human Studies:* Primary human GBM specimens for culture, PCR and Immunohistochemical analyses were obtained and utilized in accordance with a Washington University Institutional Review Board (IRB)-approved Human Studies Protocol (#201102299).

### Cell culture and reagents

U87MG cells (Catalog #HTB-14) authenticated by STR were obtained from ATCC, grown in MEM alpha (Life Technologies Cat# 12561-049) with 10% fetal bovine serum (Sigma-Aldrich Cat# F4135-500 ML), and used within 4 months of passage zero. Cells were then infected with a lentivirus expressing a fusion protein of eGFP and Firefly Luciferase (GL) to allow the cells to be visualized by fluorescence and quantified by bioluminescent imaging [23]. HBMECs were obtained from ScienCell (Catalog #1000), grown in complete endothelial media EGM-2 MV (Lonza Cat# CC-3202), and all experiments were performed between passage 6 and 10. B18 GBM lines (B18, G144) were created as described [55]. Briefly, primary GBM tumor tissue was cleaned manually of RBCs, mechanically dissociated with forceps and scalpel, and dissociated with Accutase (Sigma Cat# A6964-100 ML). Cells were then spun down, triturated gently, and plated on PLO (Sigma Cat# P4957) and Laminin (Sigma Cat# L2020) coated Primaria plates (BD Biosciences Cat# 353824). Cells were used for experiments after the fifth passage. Media is RHB-A with EGF and FGF.

### Fixed cell co-cultures

Endothelial cells were grown to 80% confluence in 6-well plates. The media was then removed and replaced with ice cold 100% methanol, placed at −20C for 20 minutes, and then washed three times with PBS before 150,000 glioblastoma cells were placed on top in their respective media.

### *In vitro* co-culture

150,000 HBMECs were plated on Matrigel (BD Biosciences Cat# 354234) using the thin gel protocol and in EGM-2 MV in 6-well plates and allowed to form tubules for 24 hours. 150,000 U87-GL cells were plated on top of the HBMEC tubules, grown together for another 48 hours, and then RNA was harvested with Trizol (Invitrogen #15596-026).

### *In vitro* angiogenesis

The *in vitro* angiogenesis assay utilized *in vitro* co-cultures as described above with the following modifications: Twenty-four hours after the addition of the U87 cells, 10x images were tiled down the middle of each well from top to bottom, stitched together in Adobe Photoshop CS4®, and all cropped to the same size. This was repeated for 48 and 72 hours at which point the images were submitted for quantification analysis to wimasis.com.

### Microarray

RNA quality was analyzed with an Agilent 2100 BioAnalyzer. Illumina HumanHT-12 v4 Expression Beadchip was performed by the Genome Technology Access Center (GTAC) according to manufacturer’s directions. Data was analyzed as described in Methods S1. The data discussed in this publication have been deposited in the NCBI’s Gene Expression Omnibus [56] and are accessible through GEO Series accession number GSE51253.

### qRT-PCR

qRT-PCR primers were designed in Primer 3 using previously described settings [57]. Primers were: GAPDH(F-TGTAGTTGAGGTCAATGAAGGG, R-ACATCGCTCAGACACCATG), CXCL1(F- GCACTGCTGCTCCTGCTCCTGGTAG, R- CGCCCATTCTTGAGTGTGGCTATGA), IL8(F- GACCACACTGCGCCAACACAGAAAT, R- CCAGTTTTCCTTGGGGTCCAGACAG), THBS1(F- CTGAGTTGGACGTCCCCATCCAAAG, R- CCACGTTGTTGTCAAGGGTGAGGAG), CXCL6(F- TGCACTTGTTTACGCGTTACGCTGAG, R- TTCCGGGTCCAGACAAACTTGCTTC), and PDE7B(F-CTGTTAAGTAGGCGGAAGTCAA, R-CGATCAGAATGCCAAATGTGTT). Thermo-Fisher qRT-PCR master mix (Thermo-Fisher Cat# AB-1166/B) was used according to manufacturer’s directions. Data was acquired on a BioRad CFX96 qPCR machine (Cat# 184-5096) and analysis was done in Microsoft Excel using the ΔΔC(t) method. RNA from 22 primary GBM specimens was obtained from the Siteman Cancer Center Tissue Procurement Core.

### Data Mining

The Oncomine Platform (Life Technologies, Ann Arbor, MI) was used for analysis and visualization. NCI’s Rembrandt Database was used for determining survival in astrocytoma patients as a function of gene expression.

### Creation of overexpression cell lines

Wild Type PDE7B or a catalytically inactive version were cloned into a vector with CMV driving cassette expression, hygromycin as a selectable marker, and flanked with PiggyBac Transposase LTRs. These plasmids were co-transfected into an existing U87-GL line and a primary GBM line, G144, with a plasmid transiently expressing PiggyBac Transposase to create stable lines as described previously [58].

### Site directed mutagenesis

The catalytically inactive version of PDE7B was created from the wild type clone of PDE7B using a site directed mutagenesis kit (Agilent Cat# 200521), as directed, with primers (F- GCTGGTTCACCCCTGGCTGGTCCACATCGTGTGC) and (R- GCACACGATGTGGACCAGCCAGGGGTGAACCAGC).

### Extreme limiting dilution assays

Extreme limiting dilution assays were performed as described previously [59]. Briefly, for each of 5 biological replicates U87-GL+PDE7B(WT) or U87-GL+PDE7B(H217Q) cells were plated at 81, 27, 9, 3, or 1 cells per well (in 12 technical replicate wells each) in tumorsphere media in 96 well plates, grown for 3 weeks, and then counted.

### Generation of intracranial xenografts

Intracranial xenografts were generated as described previously [26]. Briefly, homozygous NCR nude mice were positioned in a stereotactic frame (Stoelting) and 50,000 cells in 5 ml PBS were injected through a 27 gauge needle over 1 min at 3 mm below the dura mater, 2 mm lateral and 2 mm posterior to Bregma.

### Bioluminescence imaging

NCR nude mice bearing intracranial xenografts of U87-GL overexpressing Wild Type PDE7B or catalytically inactive control PDE7B-H217Q were injected with D-luciferin (150 µg/g; Biosynth) as previously described [23]. After anesthesia using 2.5% isoflurane, mice were imaged with a charge-coupled device camera-based bioluminescence imaging system (IVIS 50; Caliper Perkin-Elmer). Images were processed using Living Image and IgorPro Software (Version 2.50) as described. Data were expressed as total photon flux (photons/s).

### cAMP measurement

cAMP was measured by competitive immunoassay using a Correlated Enzyme Immunoassay Kit (Assay Designs) according to the manufacturer’s instructions and as previously described [60]. cAMP values were normalized to protein for each sample individually.

### The Cancer Genome Atlas Data Analysis

Gene expression data for the original 202 profiled GBM specimens (File: TCGA_unified_CORE_ClaNC840.txt, Cancer Cell) were obtained at: http://tcga-data.nci.nih.gov/docs/publications/gbm_exp/. The sample set was divided into four molecular subclasses of GBM based on signature genetic alterations: (i) *Classical,* (ii) *Mesenchymal,* (iii) *Proneural* and (iv) *Neural* [61], [62]. Subtype-characteristic centroid profiles were obtained by averaging the subset of genes defining each GBM subtype. We then explored the relationship between the four centroid profiles with PDE7B expression by pair-wise scatter plots and Pearson correlation coefficients (**Figure S4**). PDE7B gene expression was tested between subjects of different subtypes by two sample *t*-test in a pair-wise manner, and Bonferroni correction was applied to adjust the resulting raw p-values.

## Supporting Information

Figure S1 **U87 glioblastoma cells increase the in vitro angiogenesis of HBMECs.** (**A**) Representative images of day three HBMECs either in monoculture (top) or co-cultured with U87 cells (bottom). (**B**) Images after analysis by the WimTube image analysis module from wimasis.com. (**C**) Total tubule length, covered area, and total loops were quantified over areas equivalent to ∼20 fields of view in biological triplicates for HBMECs monocultures and co-cultures with U87 cells over a three day time course. Statistical significance as measured by the Student’s t-test. *<0.05, **<0.005, ***<0.0005.
(TIF)

Figure S2 **PDE4A and PDE8B expression are correlated with Survival** **in GBM.** Data from NCI’s Rembrandt database indicates that two-fold lower than median (**A**) PDE4A expression (low expression) (p-value = 0.011) and (**B**) PDE8B (p-value 3.95E-5) are correlated with worse survival compared to median (intermediate expression) levels of expression of each. In addition, two-fold higher than median expression of PDE8B (high expression) is correlated with greater survival compared to median (intermediate) expression (p-value = 0.0086) or low levels (p-value = 1.15E-6) of expression.
(TIF)

Figure S3 **Validation of PDE7B overexpression.** (**A**) qRT-PCR for PDE7B showed a 415 fold (Wild type) and 372 fold (H217Q) overexpression in U87 cells. (**B**) cAMP measurements in U87 cells grown in vitro. N = 5 (WT), N = 6 (H217Q). p-value = 0.017 by Student’s t-test. (**C**) A representative Western for PDE7B showed a ∼3 fold overexpression of PDE7B protein in cells expressing wild type PDE7B and a 1.5 fold overexpression in cells expressing the catalytically inactive H217Q form of PDE7B.
(TIF)

Figure S4 **Subtype specific expression of PDE7B.** Pair-wise scatter plots and accompanying Pearson correlation coefficients for comparisons of PDE7B expression with each GBM subtype-characteristic centroid expression profiles.
(TIF)

Methods S1 **Supplemental methods: microarray analysis.**
(DOCX)
