# Supplementary figures and images for: PDE7B Is a Novel, Prognostically Significant Mediator of Glioblastoma Growth Whose Expression Is Regulated by Endothelial Cells
Source: PLoS One. 2014 Sep 9;9(9):e107397. doi: 10.1371/journal.pone.0107397 (PMC4159344; doi:10.1371/journal.pone.0107397)

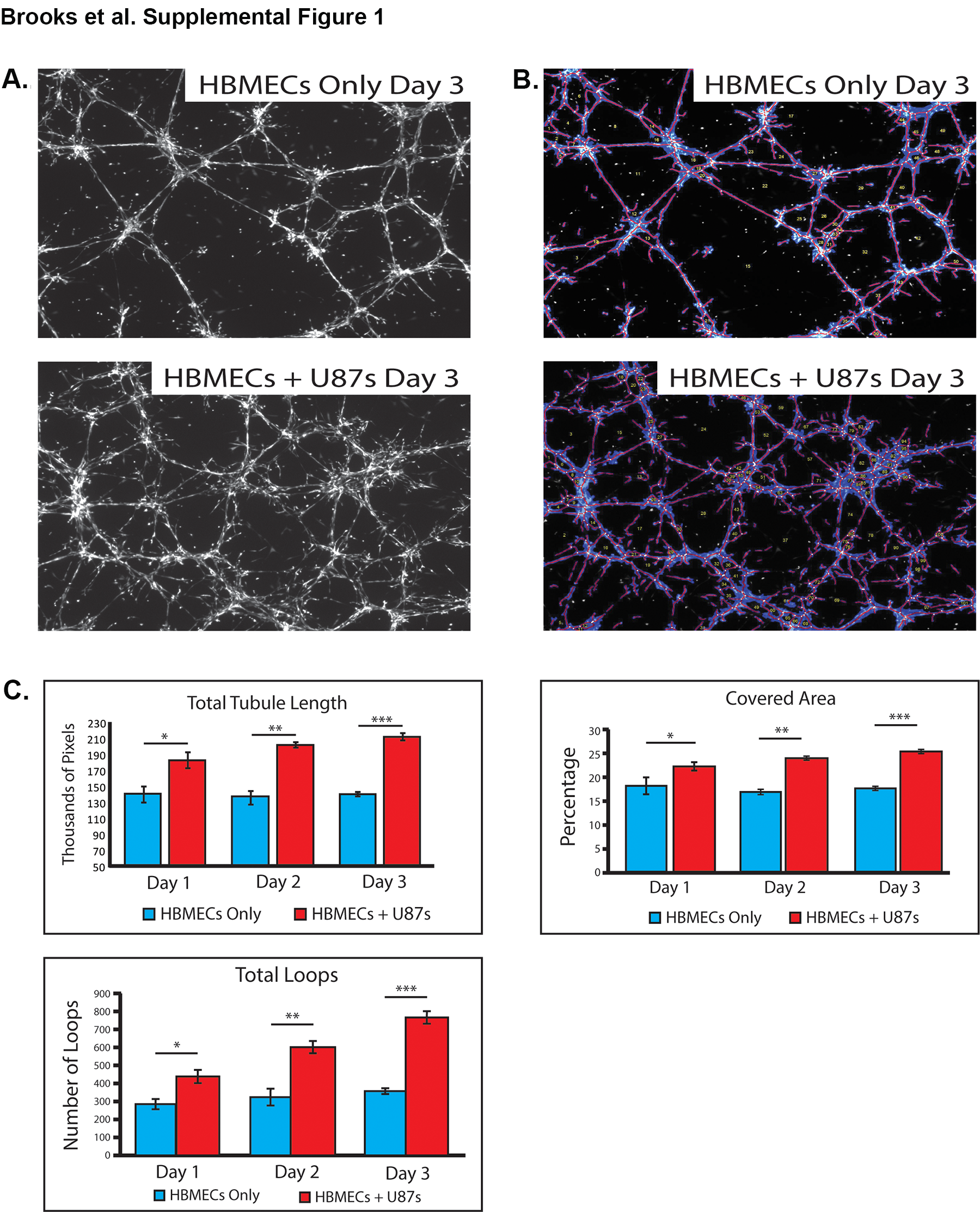

Supplement: Figure S1 — U87 glioblastoma cells increase the in vitro angiogenesis of HBMECs. (A) Representative images of day three HBMECs either in monoculture (top) or co-cultured with U87 cells (bottom). (B) Images after analysis by the WimTube image analysis module from wimasis.com. (C) Total tubule length, covered area, and total loops were quantified over areas equivalent to ∼20 fields of view in biological triplicates for HBMECs monocultures and co-cultures with U87 cells over a three day time course. Statistical significance as measured by the Student’s t-test. *<0.05, **<0.005, ***<0.0005. (TIF) [file pone.0107397.s001.tif]

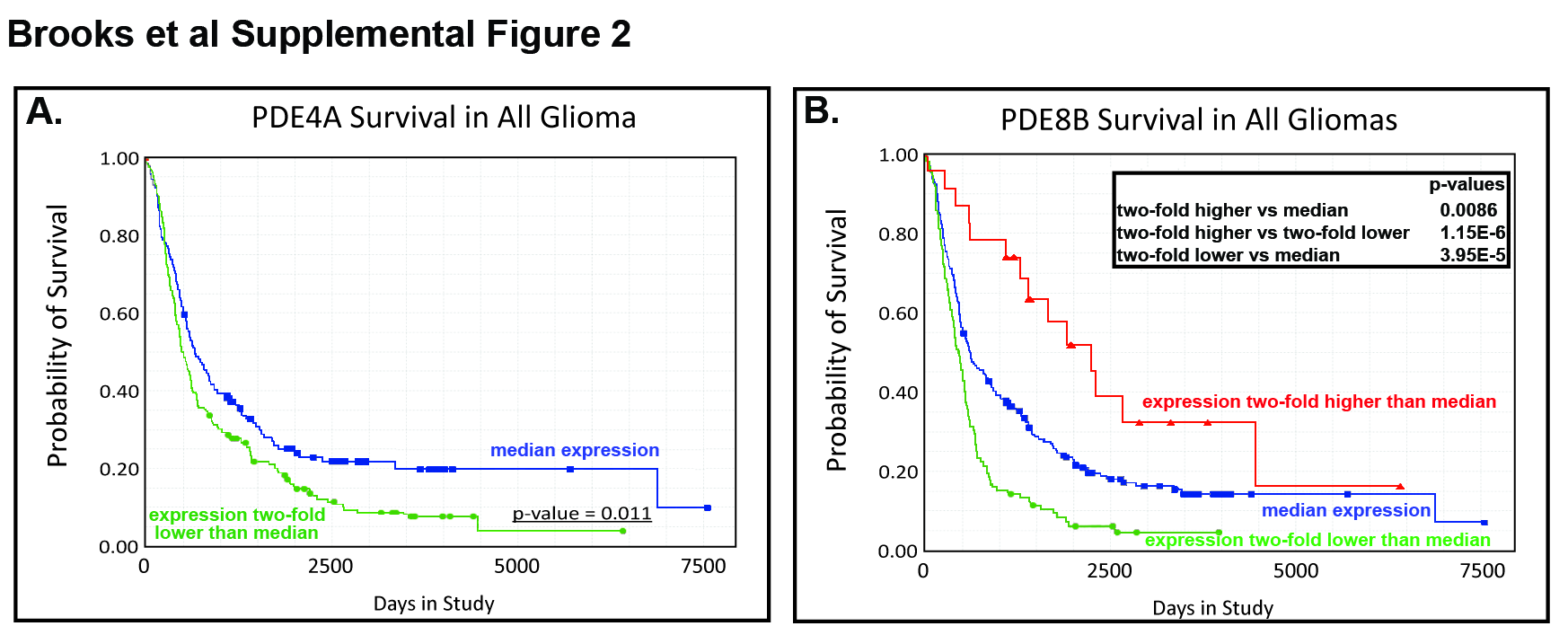

Supplement: Figure S2 — PDE4A and PDE8B expression are correlated with Survival in GBM. Data from NCI’s Rembrandt database indicates that two-fold lower than median (A) PDE4A expression (low expression) (p-value = 0.011) and (B) PDE8B (p-value 3.95E-5) are correlated with worse survival compared to median (intermediate expression) levels of expression of each. In addition, two-fold higher than median expression of PDE8B (high expression) is correlated with greater survival compared to median (intermediate) expression (p-value = 0.0086) or low levels (p-value = 1.15E-6) of expression. (TIF) [file pone.0107397.s002.tif]

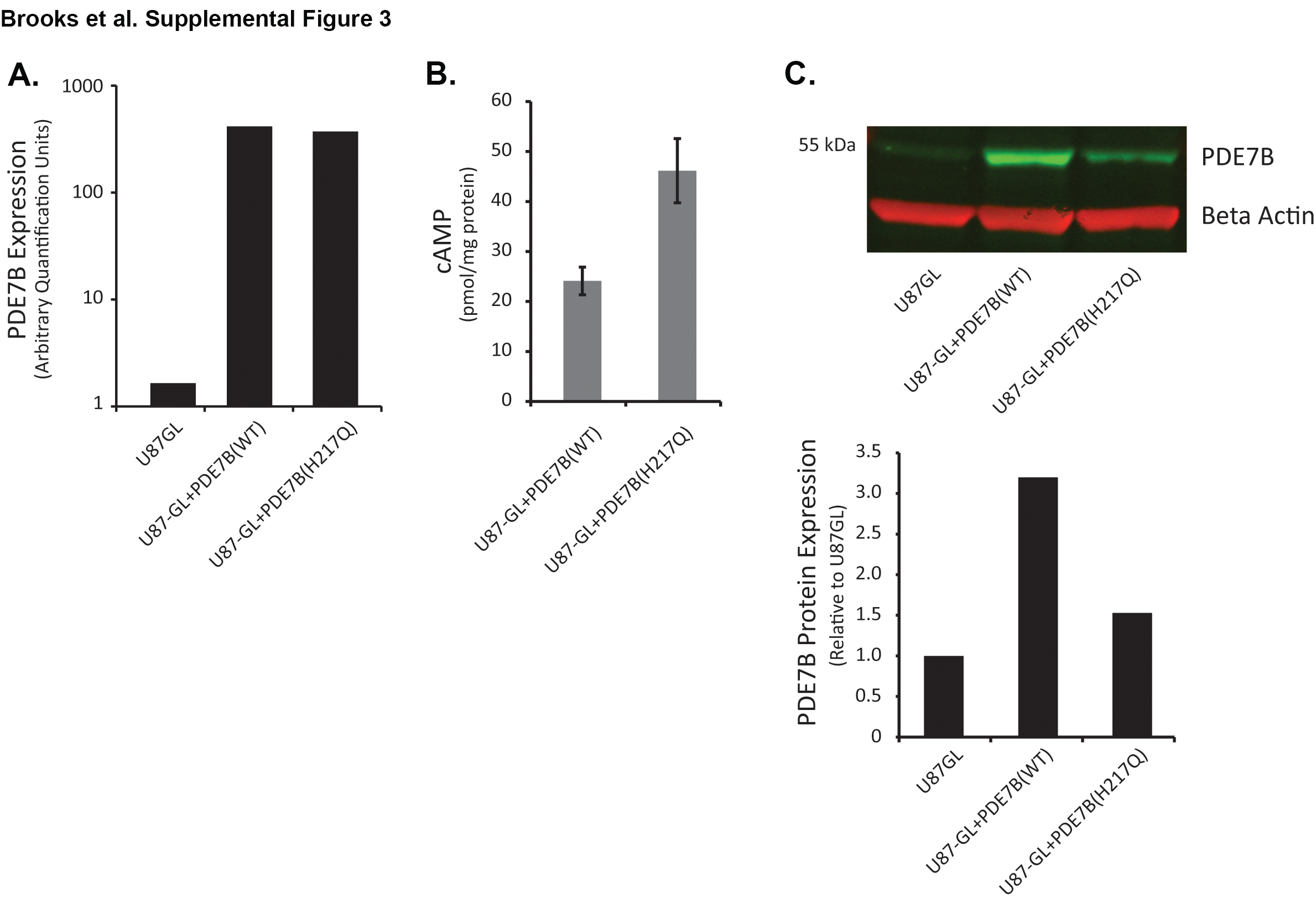

Supplement: Figure S3 — Validation of PDE7B overexpression. (A) qRT-PCR for PDE7B showed a 415 fold (Wild type) and 372 fold (H217Q) overexpression in U87 cells. (B) cAMP measurements in U87 cells grown in vitro. N = 5 (WT), N = 6 (H217Q). p-value = 0.017 by Student’s t-test. (C) A representative Western for PDE7B showed a ∼3 fold overexpression of PDE7B protein in cells expressing wild type PDE7B and a 1.5 fold overexpression in cells expressing the catalytically inactive H217Q form of PDE7B. (TIF) [file pone.0107397.s003.tif]

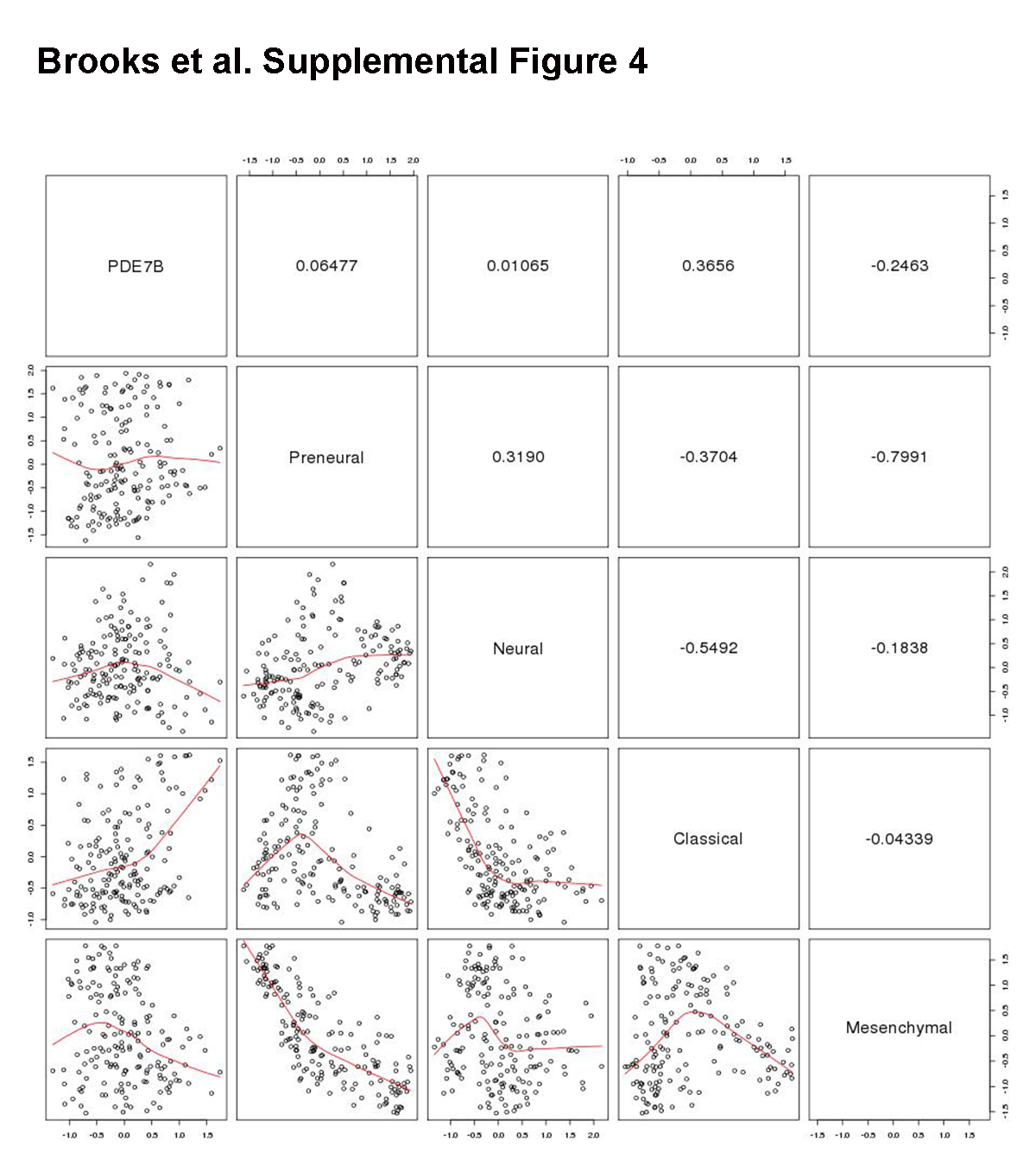

Supplement: Figure S4 — Subtype specific expression of PDE7B. Pair-wise scatter plots and accompanying Pearson correlation coefficients for comparisons of PDE7B expression with each GBM subtype-characteristic centroid expression profiles. (TIF) [file pone.0107397.s004.tif]
