# Supplementary material for: PDE7B Is a Novel, Prognostically Significant Mediator of Glioblastoma Growth Whose Expression Is Regulated by Endothelial Cells
Source: PLoS One. 2014 Sep 9;9(9):e107397. doi: 10.1371/journal.pone.0107397 (PMC4159344; doi:10.1371/journal.pone.0107397)
Supplement: Methods S1 — Supplemental methods: microarray analysis. (DOCX) [file pone.0107397.s005.docx]

**Supplemental Methods:**

Microarray analysis

Starting with the quartile normalized Illumina Beadarray data from the standard Illumina analysis, a perl script loaded the data for three biological replicates of U87 monoculture, HBMEC monoculture, and U87 plus HBMEC co-culture. Data was filtered for genes that had greater than a baseline expression value with a likelihood of expression p-value of less than 0.05 (as determined by the Illumina software). For each test computational dataset to be created (0.1% U87/99.9% HBMEC for example), and for each gene in the dataset, the value for biological replicate 1 of the U87 data was multiplied by 0.001 (for example) and added to the value for biological replicate 1 of the HBMEC data after it was multiplied by 0.999. This was repeated for the other two biological replicates to give 3 new computationally derived replicates and then repeated for all of the genes. After each of these computational datasets was complete, a Pearson correlation was calculated between of all the genes for biological replicate 1 of the co-culture dataset versus all the genes of the computationally derived biological replicate 1. This was repeated for all three replicates, and then these three Pearson correlations were averaged and recorded. Figure 1B is a plot of all the different computationally derived datasets versus the corresponding average Pearson correlations.

Once the computational dataset was created, genes without any expression (average of the three biological replicates < 30) in any of the samples were removed from the analysis leaving 14,712 genes. Next, a Student’s t-test was performed between the 3 computational replicates and the three co-culture replicates. Further filtering was performed to leave only those genes that had a fold change greater than 2 fold (up or down) between the computational dataset and the co-culture dataset. Benjamini-Hochberg multiple hypothesis correction was then performed and changes in expression of 45 genes remained statistically significant.
